# QSAR analysis on tacrine-related acetylcholinesterase inhibitors

**DOI:** 10.1186/s12929-014-0084-0

**Published:** 2014-09-20

**Authors:** Kai Y Wong, Andrew G Mercader, Laura M Saavedra, Bahareh Honarparvar, Gustavo P Romanelli, Pablo R Duchowicz

**Affiliations:** Department of Chemical Engineering, South Kensington Campus, Imperial College London, SW7 2AZ London, UK; Instituto de Investigaciones Fisicoquímicas Teóricas y Aplicadas INIFTA (UNLP, CCT La Plata-CONICET), Diag. 113 y 64, Sucursal 4, C.C. 16, 1900 La Plata, Argentina; Centro de Investigación y Desarrollo en Ciencias Aplicadas “Dr. J.J. Ronco” (CINDECA), Departamento de Química, Facultad de Ciencias Exactas, UNLP-CCT-CONICET, Calle 47N° 257, B1900AJK La Plata, Argentina; School of Pharmacy and Pharmacology, University of KwaZulu-Natal, 4001 Durban, South Africa

**Keywords:** Alzheimer’s disease, Acetylcholinesterase Inhibitor, QSAR theory, Tacrine, Validation

## Abstract

**Background:**

The evaluation of the clinical effects of Tacrine has shown efficacy in delaying the deterioration of the symptoms of Alzheimer’s disease, while confirming the adverse events consisting mainly in the elevated liver transaminase levels. The study of tacrine analogs presents a continuous interest, and for this reason we establish Quantitative Structure-Activity Relationships on their Acetylcholinesterase inhibitory activity.

**Results:**

Ten groups of new developed Tacrine-related inhibitors are explored, which have been experimentally measured in different biochemical conditions and AChE sources. The number of included descriptors in the structure-activity relationship is characterized by ‘Rule of Thumb’. The 1502 applied molecular descriptors could provide the best linear models for the selected Alzheimer’s data base and the best QSAR model is reported for the considered data sets.

**Conclusion:**

The QSAR models developed in this work have a satisfactory predictive ability, and are obtained by selecting the most representative molecular descriptors of the chemical structure, represented through more than a thousand of constitutional, topological, geometrical, quantum-mechanical and electronic descriptor types.

**Electronic supplementary material:**

The online version of this article (doi:10.1186/s12929-014-0084-0) contains supplementary material, which is available to authorized users.

## Background

Alzheimer’s Disease (AD) is a neurodegenerative process characterized by a progressive memory loss, decline in language skills and other cognitive abilities [1]. It is common among the elderly, affecting around 7% of the population above 65 years old [2]. Currently, this is an incurable disease without an effective therapeutic approach [3]. However, there exists a palliative strategy which enhances cholinergic transmission, as defined by the cholinergic hypothesis [4,5]. Patients with AD experience a selective loss of cholinergic neurons in the brain and decreasing levels of Acetylcholine, a neurotransmitter [6]. The Acetylcholinesterase enzyme (AChE) is responsible for terminating impulse signaling at cholinergic synapses by catalyzing the hydrolysis of Acetylcholine [7].

Tacrine (Additional file 1, compound 1) is an example of Acetylcholinesterase inhibitor (AChEI). It has been synthesized more than forty years ago, and in 1993, it has become the first drug to be approved by the US Food and Drug Administration as a form of treatment for AD [8]. Although Tacrine is the oldest palliative drug designed based on the cholinergic hypothesis, new Tacrine derivatives are still being designed to treat AD. It has been experimentally demonstrated that bis-Tacrine congeners display enhanced inhibitory activity towards AChE compared to Tacrine [9-11].

Quantitative Structure-Activity Relationships (QSAR) are mathematical frameworks which link molecular structures of compounds to their biological activities in a quantitative manner [12-14]. Although these tend to be statistical models rather than deterministic ones based on fundamental physical laws, the QSAR approach highlights parallelisms between structure and potency. Such knowledge allows researchers to pick out the most promising structures from a much larger pool of potential compounds, making drug design more rational by reducing the number of expensive, time consuming experiments.

Among the QSAR research carried out on Tacrine derivatives, in 2006 Akula et al. [15] have published 3D-QSAR studies on bis-tacrine compounds by using molecular docking scores, in addition to Comparative Molecular Field Analysis (CoMFA). The Sybyl [16] and Mopac [17] programs are both used in the optimization of structures and molecular alignment. The docking scores are used as molecular descriptors along with the steric and electrostatic field values obtained from CoMFA, and 16 molecules are set aside in the training set. The structure-activity model is validated on a test set having 3 molecules. The proposed models provide insights into the inhibitory mechanism and could help designing new inhibitors. In another study of the same year, Fernández et al. [18] have applied Bayesian-Regularized Genetic Neural Networks (BRGNNs) to 136 Tacrine analogues. Here, the Bayesian-regularization avoids overtraining, while the Genetic Algorithm (GA) approach allows exploring an ample pool of 3D-descriptors generated by the Dragon software [19]. The resulting model is evaluated by averaging multiple validation sets generated as members of diverse-training set Neural Network Ensembles (NNEs). When considering 40 assembled members, the NNE provides reliable statistics.

In 2007, Jung et al. [20] have built QSAR models using variable selections based on Multivariable Linear Regression (MLR) approaches: Genetic Algorithms (GA) and Simulated Annealing (SA). The authors compile a set of 80 structurally heterogeneous compounds composed of 11H-indeno-[1,2-b]-quinolin-10-ylamine derivatives, thio-pyranoquinolines, pyranoquinolines and benzonaphthyridines, tacrine-E2020 hybrids, bis-tacrine congeners, and tacrine-hurprine heterodimers, placing 68 Tacrine derivatives in the training set and leaving 12 in the test set. The molecules are geometrically optimized using the Titan Pro software [21], and their molecular descriptors are calculated using PreADME on the web [22] and BioMedCAChe [23]. The best model is obtained by SA-MLR with greater explanatory and prediction capability. The authors suggest important roles for hydrophobic and electrostatic interactions on increasing the structure’s AChE activity. They also argue opposite effects for hydrophilic and topological features of molecules.

Saracoglu et al. [24] have performed in 2008 QSAR analyses of AChEIs related to Tacrine and 11 H-Indeno-[1,2-b]-quinolin-10-ylamine tetracyclic Tacrine analogues. The Electron-Topological Method (ETM) is applied with the ETM software [25] on a training set of 44 compounds, which we consider it as a valuable QSAR tool as this technique takes into account both geometrical and electronic characteristics of the molecules. Based on pharmacophores and anti-pharmacophores calculated as sub-matrices containing spatial and quantum chemistry characteristics, a system for the activity prognostication is developed. Some molecular fragments specific for active and inactive compounds are also revealed.

In a recent work of 2012, Chen et al. [26] have studied multi-target-directed AChEIs of Tacrine-Nimodipine dihydropyridines. They establish 3D-QSAR models using CoMFA and Comparative Molecular Similarity Index Analysis (CoMSIA) methods. The compounds employed are very potent and selective AChEIs, and show an excellent neuroprotective profile and a moderate Ca^2+^ channel blockage effect. A training set of 60 compounds is used, and the resulting models are validated on a test set of 12 compounds. The structures of the investigated ligands are built and optimized using Sybyl [16], while the lowest energy structures are used during the alignment. The partial atomic charges required to estimate the electrostatic interaction are computed by semi-empirical molecular orbital methods using Mopac [17] with an AM1 Hamiltonian. For the CoMSIA approach, descriptors of five physicochemical field properties are used to correlate with changes of ligands affinities, which explicitly define hydrophobic, hydrogen-bond donor and acceptor descriptors (in addition to the steric and electrostatic fields used in CoMFA).

It is notable that most published QSAR studies that analyze a higher number of Tacrine compounds involve inhibitory activities measured on AChE samples extracted from different sources [18,20]. In this work, we complement the research performed previously by considering quantitative structure-activity relationships for ten homogeneous classes of new developed Tacrine compounds, each class measured under different biochemical conditions and extracted from a different AChE source. Therefore, present QSAR study is specific for each data set. In such a situation of having a scarce amount of experimental data for each AChEI group, the combination of the MLR technique and the ‘Rule of Thumb’ [27] has proven to be appropriate for developing predictive multi-parametric QSAR models [28-31].

## Methods

### Experimental AChE inhibitory activity

The ten classes of new synthesized Tacrine-like inhibitors have been measured by varying the temperature, incubation time and number of tested concentrations, and using different sources of AChE. AChE inhibitory activity is spectrophotometrically evaluated by the Ellman method [32], using AChE from bovine (bAChE) or human erythrocytes (hAChE) and Acetylthiocholine iodide (0.53 or 0.13 mM for bAChE and hAChE, respectively) as substrate. AChE activity in *Electrophorus electricus* (EeAChE) is measured following the spectrophotometric Rappaport method [33] using purified AChE from Electric eel ( E. electricus) and Acetylcholine chloride (29.5 mM) as substrate. The inhibitory activity is presented as half of the maximal inhibitory concentration in molar units (log_10_(*IC*_50_)).

The data sets of Additional file 1 are composed as follows: Data set A includes 7 pyrano[2,3-*b*]quinolines, 6 benzonaphtyridines and Tacrine, as in reference [34], with measured values of bAChE (Additional file 2: Table S1-S7). Data set B includes 2 hydrochlorides of 6-chlorotacrine and tricyclic esters, Propidium iodide, 10 dihydrochlorides of the pyrano[3,2-*c*]quinoline-6-chlorotacrine and 6-chlorotacrine, with values of bAChE and hAChE (Additional file 2: Table S2) [35]. Data set C consists on 17 Tacrine-dihydropyridine hybrids, Tacrine and Propidium, presenting measured values of EeAChE and hAChE (Additional file 2: Table S3) [36]. Data set D is composed of 8 donepezil-Tacrine hybrid related derivatives, Tacrine and Donepezil, having measured values of bAChE (Additional file 2: Table S4) [37]. Data set E consists on 12 Tacrine-based dimers, *bis*(7)-Tacrine and Tacrine, presenting hAChE measured values (Additional file 2: Table S5) [38]. Data set F includes 12 Tacrine analogs from highly substituted 2-aminopyridine-3-carbonitriles and Tacrine, having measured values of bAChE and EeAChE (Additional file 2: Table S6) [39]. Data set G contains 18 Tacrine-based dual binding site Acetylcholinesterase inhibitors, Tacrine, 6-chloroTacrine, Propidium iodide and Donepezil, with known values of hAChE (Additional file 2: Table S7) [40].

### Molecular descriptors

The molecular structures are drawn with the aid of the “Add Hydrogen and Model Build” modulus of HyperChem 6.03 for Windows [41]. The compounds are firstly pre-optimized with the Molecular Mechanics Force Field (MM+) procedure included in HyperChem, and the resulting geometries are further refined by means of the Semiempirical Method PM3 (Parametric Method-3) using the Polak-Ribiere algorithm and a gradient norm limit of 0.01 kcal.Å^−1^. We keep the R-configuration for all the molecules having a chiral Carbon atom when such configuration detail is not specified in the original data.

All the molecular descriptors are computed using the software Dragon [19]. This well-known descriptors database includes descriptor families such as: 0D-descriptors: constitutional indices; 1D-descriptors: functional group counts, atom-centred fragments, empirical descriptors, molecular properties; 2D-descriptors: topological indices, walk and path counts, connectivity indices, information indices, 2D-autocorrelations, Burden eigenvalues, Galvez topological charge indices; and 3D-descriptors: aromaticity indices, Randic molecular profiles, geometrical indices, radial distribution functions, 3D-MoRSE (3D-Molecular Representation of Structure based on Electron diffraction) descriptors, WHIM (Weighted Holistic Invariant Molecular) descriptors, GETAWAY (Geometry, Topology and Atoms-Weighted AssemblY) descriptors and charge indices [42]. Finally, five quantum-chemical descriptors not provided by the Dragon program are added to the pool: molecular dipole moment, total energy, homo-lumo energy, and homo-lumo gap (∆_homo-lumo_). The resulting pool consists on *D* = 1502 structural descriptors.

### Linear model search

In past years, the MLR technique has proved to be a multidisciplinary technique of valuable applicability for establishing predictive QSAR models by different research groups, by performing an exhaustive analysis of a pool containing a great number of structural descriptors [19,43]. Linear models are more general and can transparently reveal the effect on the activity being modeled when including/excluding molecular descriptors in the equation, thus making it possible to suggest cause/effect relationships by means of simple parallelisms. The main advantage of developing linear regression models, when compared to non-linear models, is the fact that linear models suffer in a lesser extend from the over-fitting (over-training) problem [44,45] because the MLR technique does not involve too many optimization parameters during the model building, just the regression coefficient for each molecular descriptor. Therefore, the MLR is considered as the best choice of descriptor selection method when few experimental activity values are available, as it is the case in present study: few data are available for each data set A-G.

It is our purpose to search the set **D** having *D* descriptors, for an optimal subset **d** of *d < <D* ones with minimum standard deviation (*S*), by means of the MLR technique. More precisely, we want to obtain the global minimum of *S*(**d**) where **d** is a point in a space of *D*!/(*D-d*)!*d*! ones. Each point is a possible model of *d* descriptors as discussed below. Taking into account that a Full Search (FS) of optimal variables is impractical because it requires *D*!/(*D-d*)!*d*! linear regressions, some time ago we have proposed the Replacement Method (RM) [46-51] that produces linear regression QSAR models that are quite close to the FS ones with much less computational work. The RM approaches the minimum of *S* by judiciously taking into account the relative errors of the coefficients of the least-squares model given by a set of *d* descriptors **d** = {*X*_1_, *X*_2_, …, *X*_*d*_}.

The procedure of the RM technique is as follows: choose *d* descriptors {*X*_1_, *X*_2_, …, *X*_*d*_} at random and do a linear regression. Choose one of the descriptors of this set, say X_*i*_, and replace it by each of the *D* descriptors of the pool (except itself) keeping the best resulting set. Since one can start replacing any of the *d* descriptors in the initial model, then a regression equation with *d* variables has *d* possible paths to achieve the final result; for example, the choice above will develop into path *i*. Next, choose the variable with greatest relative error in its coefficient (except the one replaced in the previous step) and replace it with all the *D* descriptors (except itself) keeping again the best set. Replace all the remaining variables in the same way by passing those replaced in previous steps. When finishing, start again with the variable having greatest relative error in the coefficient and repeat the whole process. Repeat this process as many times as necessary until the set of descriptors remains unchanged. At the end, we have the best model for the path *i*. Proceed in exactly the same way for all possible paths *i* = 1, 2,…, *d*, compare the resulting models, and keep the best one. Our numerical experiments show that in this way one obtains a model almost as good as the best one with much less than *D*!/(*D-d*)!*d*! linear regressions when this combinatorial number is large. The RM gives models with better statistical parameters than the Forward Stepwise Regression procedure [52] and variants of the more elaborated Genetic Algorithms [53].

### Model validation

The design of a properly validated model constitutes the most important step for every QSAR analysis, in order to generate predictive models that involve general applicability and that are not limited to only function correlatively. The Cross-Validation technique of Leave-One-Out (loo) is practiced [54]. The parameters ${R}_{loo}^2$ and *S*_*loo*_ (square of the correlation coefficient and standard deviation of Leave-One-Out) measure the stability of the model upon exclusion of molecules. According to the literature, ${R}_{loo}^2$ should be greater than 0.5 for a validated model.

The Y-Randomization procedure [55] is also applied in order to verify that the model is robust. This technique consists on scrambling the experimental property values in such a way that they do not correspond to the respective compounds. After analyzing 1000 cases of Y-Randomization, the standard deviation obtained (S^rand^) has to be a poorer value than the one found by considering the true calibration (*S*).

Finally, we also apply the standard practice that consists on omitting from the complete molecular set some compounds which constitute the ‘test set’, denoted here as ‘test’. The main purpose of performing such a splitting is to assess whether the QSAR found have predictive capability for estimating the activity values on the independent test set compounds, that are not involved during the model fitting using the ‘training set’ compounds (denoted as ‘train’). We select the molecules composing the training and test series as a previous step to the model search, and this is done in such a way that both sets share similar qualitative structure–property characteristics.

## Results and discussion

The application of the RM variable subset selection method on the total pool with *D* = 1502 molecular descriptors leads to the best linear models for the Alzheimer’s data sets. In order to determine the number of descriptors to be included in the structure-activity relationship, we consider the ‘Rule of Thumb’, which states that at least 5 or 6 training set molecules (*N*) should be present for each fitting parameter. In the following we present the best QSAR found for data sets A-G.

Data set A (bAChE):

1 $$ \begin{array}{l} \log {}_{10}I{C}_{50}=1.345\left(\pm 2\right)-1.051\left(\pm 0.3\right)\cdot Mor06v-44.823\left(\pm 10\right)\cdot G1u\\ {}N=12,\kern0.5em  range=\left[-6.886,-4.678\right],\kern0.5em R=0.90,\kern0.5em S=0.29,\kern0.5em F=18.36,\kern0.5em p<{10}^{\hbox{-} 4},\kern0.5em \\ {}{R}_{ij}^{\max }=0.43,\kern0.5em \mathrm{o}\left(>2.S\right)=0,\kern0.5em {R}_{loo}=0.84,\kern0.5em {S}_{loo}=0.39,\kern0.5em {S}^{rand}=0.31\end{array} $$

Data set B (bAChE):

2 $$ \begin{array}{l} \log {}_{10}I{C}_{50}=-7.244\left(\pm 0.4\right)+6.741\left(\pm 0.4\right)\cdot GATS2e-4.476\left(\pm 0.4\right)\cdot R5e\\ {}N=12,\kern0.5em  range=\left[-8.242,-5.000\right],\kern0.5em R=0.98,\kern0.5em S=0.23,\kern0.5em F=143.01,\kern0.5em p<{10}^{\hbox{-} 4},\ \\ {}{R}_{ij}^{\max }=0.46,\kern0.5em \mathrm{o}\left(>2.S\right)=0,\kern0.5em {R}_{loo}=0.96,\kern0.5em {S}_{loo}=0.37,\kern0.5em {S}^{rand}=0.30\end{array} $$

Data set B (hAChE):

3 $$ \begin{array}{l} \log {}_{10}I{C}_{50}=-18.159\left(\pm 0.8\right)+101.916\left(\pm 6\right)\cdot Qmean+35.567\left(\pm 6\right)\cdot R3{m}^{+}\\ {}N=11,\kern0.5em  range=\left[-8.153,-4.491\right],\kern0.5em R=0.99,\kern0.5em S=0.22,\kern0.5em F=170.55,\kern0.5em p<{10}^{\hbox{-} 4},\ \\ {}{R}_{ij}^{\max }=0.61,\kern0.5em \mathrm{o}\left(>2.S\right)=0,\kern0.5em {R}_{loo}=0.98,\kern0.5em {S}_{loo}=0.27,\kern0.5em {S}^{rand}=0.27\end{array} $$

Data set C (EeAChE):

4 $$ \begin{array}{l} \log {}_{10}I{C}_{50}=-2.611\left(\pm 0.3\right)-83.235\left(\pm 8\right)\cdot R5{e}^{+}-1.592\left(\pm 0.1\right)\cdot nNHR\\ {}N=16,\kern0.5em  range=\left[-7.284,-4.155\right],\kern0.5em R=0.97,\kern0.5em S=0.24,\kern0.5em F=109.06,\kern0.5em p<{10}^{\hbox{-} 4},\ \\ {}{R}_{ij}^{\max }=0.25,\kern0.5em \mathrm{o}\left(>2.S\right)=0,\kern0.5em {R}_{loo}=0.95,\kern0.5em {S}_{loo}=0.32,\kern0.5em {S}^{rand}=0.50\end{array} $$

Data set C (hAChE):

5 $$ \begin{array}{l} \log {}_{10}I{C}_{50}=16.021\left(\pm 4\right)-52.448\left(\pm 10\right)\cdot X1A\\ {}N=9,\kern0.5em  range=\left[-6.959,-5.553\right],\kern0.5em R=0.90,\kern0.5em S=0.24,\kern0.5em F=28.44,\kern0.5em p<{10}^{\hbox{-} 4},\ \\ {}{R}_{ij}^{\max }=0,\kern0.5em \mathrm{o}\left(>2.S\right)=0,\kern0.5em {R}_{loo}=0.84,\kern0.5em {S}_{loo}=0.30,\kern0.5em {S}^{rand}=0.25\end{array} $$

Data set D (bAChE):

6 $$ \begin{array}{l} \log {}_{10}I{C}_{50}=-5.510\left(\pm 0.1\right)-1.263\left(\pm 0.1\right)\cdot Mor09u-32.160\left(\pm 2.0\right)\cdot R2{m}^{+}\\ {}N=9,\kern0.5em  range=\left[-8.553,-5.522\right],\kern0.5em R=0.98,\kern0.5em S=0.18,\kern0.5em F=98.81,\kern0.5em p<{10}^{\hbox{-} 4},\ \\ {}{R}_{ij}^{\max }=0.30,\kern0.5em \mathrm{o}\left(>2.S\right)=0,\kern0.5em {R}_{loo}=0.95,\kern0.5em {S}_{loo}=0.33,\kern0.5em {S}^{rand}=0.26\end{array} $$

Data set E (hAChE):

7 $$ \begin{array}{l} \log {}_{10}I{C}_{50}=5.385\left(\pm 2\right)-6.669\left(\pm 1\right)\cdot BELm2-2.697\left(\pm 0.3\right)\cdot Mor27e\\ {}N=12,\kern0.5em  range=\left[-9.092,-6.342\right],\kern0.5em R=0.96,\kern0.5em S=0.29,\kern0.5em F=51.70,\kern0.5em p<{10}^{\hbox{-} 4},\\ {}{R}_{ij}^{\max }=0.046,\kern0.5em \mathrm{o}\left(>2.S\right)=0,\kern0.5em {R}_{loo}=0.92,\kern0.5em {S}_{loo}=0.39,\kern0.5em {S}^{rand}=0.46\end{array} $$

Data set F (bAChE):

8 $$ \begin{array}{l} \log {}_{10}I{C}_{50}=-17.546\left(\pm 2\right)+1.510\left(\pm 0.2\right)\cdot AMW+0.247\left(\pm 0.2\right)\cdot MEcc\\ {}N=11,\kern0.5em  range=\left[-7.000,-4.000\right],\kern0.5em R=0.98,\kern0.5em S=0.25,\kern0.5em F=21.33,\kern0.5em p<{10}^{\hbox{-} 4},\ \\ {}{R}_{ij}^{\max }=0.13,\kern0.5em \mathrm{o}\left(>2.S\right)=0,\kern0.5em {R}_{loo}=0.96,\kern0.5em {S}_{loo}=0.36,\kern0.5em {S}^{rand}=0.39\end{array} $$

Data set F (EeAChE):

9 $$ \begin{array}{l} \log {}_{10}I{C}_{50}=-15.372\left(\pm 1\right)+13.386\left(\pm 2\right)\cdot E2u+18.306\left(\pm 1\right)\cdot HATS6m\\ {}N=11,\kern0.5em  range=\left[-7.854,-4.523\right],\kern0.5em R=0.99,\kern0.5em S=0.19,\kern0.5em F=150.88,\kern0.5em p<{10}^{\hbox{-} 4},\\ {}{R}_{ij}^{\max }=0.66,\kern0.5em \mathrm{o}\left(>2.S\right)=0,\kern0.5em {R}_{loo}=0.97,\kern0.5em {S}_{loo}=0.28,\kern0.5em {S}^{rand}=0.34\end{array} $$

Data set G (hAChE):

10 $$ \begin{array}{l} \log {}_{10}I{C}_{50}=66.887\left(\pm 6\right)+0.014\left(\pm 0.0009\right)\cdot MPC09-78.669\left(\pm 6\right)\cdot MATS1m\\ {}-6.087\left(\pm 0.3\right)\cdot RDF020m\\ {}N=19,\kern0.5em  range=\left[-9.569,-5.000\right],\kern0.5em R=0.98,\kern0.5em S=0.21,\kern0.5em F=131.81,\kern0.5em p<{10}^{\hbox{-} 4},\\ {}{R}_{ij}^{\max }=0.65,\kern0.5em \mathrm{o}\left(>2.S\right)=3,\kern0.5em {R}_{loo}=0.97,\kern0.5em {S}_{loo}=0.29,\kern0.5em {S}^{rand}=0.53\end{array} $$

In these equations, *range* stands for the range of experimental activity of the training set, *R* is the correlation coefficient, *S* is the standard deviation of the model, *F* is the Fisher parameter, *p* is the significance of the model, ${R}_{ij}^{\max }$ denotes the maximum intercorrelation coefficient between descriptors, o(>2.*S*) indicates the number of outlier molecules having a residual greater than two times the standard deviation, and *R*_*loo*_ and *S*_*loo*_ are the correlation coefficient and standard deviation obtained with the loo technique, respectively.

In most cases, it is appreciated that a single descriptor does not achieve enough accuracy for predicting the AD activities, while models based on two- or three- descriptors are acceptable for the number of training molecules involved. When we plot the QSAR predicted log_10_*IC*_50_ inhibitory activities as function of experimental values for each data set (**A-G**) in Additional file 3, a straight line trend is observed. It is also appreciated that Eqs. 1–10 predict reasonably well the experimental activities of the molecules that are members of the test set, and thus the models established tend to behave as predictive. In addition, both parameters *R*_*loo*_ and *S*_*loo*_ measure the stability of the developed QSAR upon inclusion/exclusion of compounds, and according to the specialized literature, ${R}_{loo}^2$ must be greater than 0.50 for obtaining a validated model [54].

Dispersion plots of residuals (residuals as function of predicted activities) for each QSAR are provided in Additional file 4: Figure S1A up to S1G with the purpose of demonstrating the validity of these MLR equations. Although some outliers are detected in some plots, having residuals exceeding the 2.*S* value, we decide to derive general models having applicability to any biomolecule without restrictions, and so we do not remove such molecules from the training set.

The molecular descriptors appearing in Eqs. 1–10 are of different types and are briefly described in Table 1. We provide their numerical values in Additional file 2: Tables S1-S7. Constitutional descriptors are 0D-descriptors, independent from the molecular connectivity and conformation; they are the most simple and commonly used descriptors, reflecting the molecular composition of a compound. The topological indices derived from the Chemical Graph Theory [56] are obtained with a graph representation of the molecule, that is to say, its planar image, and provide only information on the structural composition and connectivity but nothing about its three dimensional or stereochemical aspects. In a molecular graph, the vertices are atoms weighted with different physicochemical properties such as mass, polarity, electronegativity, charge, etc. The 3D-MoRSE (3D-Molecule Representation of Structure based on Electron diffraction) descriptors [57] provide 3D-information from the molecular structure using a molecular transform derived from an equation used in electron diffraction studies. Several atomic properties can be taken into account, thus giving high flexibility to this representation of a molecule.

**Table 1:** **Notation and brief description of the molecular descriptors involved in the QSAR models for AD**

**Molecular descriptor**	**Type**	**Description**
*Mor*06*v*	3D-MoRSE	3D-MoRSE - signal 06/weighted by atomic van der Waals volumes
*Mor*09u		3D-MoRSE - signal 09/unweighted
*Mor*27*e*		3D-MoRSE - signal 27/weighted by atomic Sanderson electronegativities
*G*1*u*	WHIM	1st component symmetry directional WHIM index/unweighted
*Eu*2		2nd component accessibility directional WHIM index/unweighted
*GATS*2*e*	2D- Autocorrelations	Geary autocorrelation of lag 2/weighted by atomic Sanderson electronegativities
*MATS*1*m*		Moran autocorrelation of lag 1/weighted by atomic masses
*R*5*e*	GETAWAY	R autocorrelation of lag 5/weighted by atomic Sanderson electronegativities
*HATS*6*m*		leverage-weighted autocorrelation of lag 6/weighted by atomic masses
*R*3*m* ^+^		R maximal autocorrelation of lag 3/weighted by atomic masses
*R*2*m* ^+^		R maximal autocorrelation of lag 2/weighted by atomic masses
*R*5*e* ^+^		R maximal autocorrelation of lag 5/weighted by atomic Sanderson electronegativities
*nNHR*	Constitutional	number of secondary amines (aliphatic)
*AMW*		average molecular weight
*X*1*A*	Topological	average connectivity index chi-1
*MPC*09		molecular path count of order 09
*Qmean*	Charge	mean absolute charge (charge polarization)
*BELm*2	BCUT	lowest eigenvalue number 2 of Burden matrix/weighted by atomic masses
*MEcc*	Geometrical	molecular eccentricity
*RDF*020*m*	RDF	Radial Distribution Function - 2.0/weighted by atomic masses

WHIM (Weighted Holistic Invariant Molecular Descriptors) descriptors are based on statistical indices calculated on the projections of atoms along principal axes [58]. The aim is to capture 3D-information regarding size, shape, symmetry and atom distributions with respect to invariant reference frames. In order to calculate them, a weighted covariance matrix is obtained from different weighting schemes for the atoms. The different structural variables introduced by Broto, Moreau, and Geary [59] account for bi-dimensional autocorrelations between atoms pairs in the molecule, and are defined in order to reflect the contribution of a considered atomic property to the experimental observations under investigation. These indices can be readily calculated, *i.e.*: by summing products of atomic weights (employing atomic properties such as atomic polarizabilities, molecular volumes, etc.) of the terminal atoms of all the paths of a prescribed length.

The GETAWAY (GEometry, Topology, and Atom-Weights AssemblY) type of descriptors [60] have been designed with the main purpose of matching the 3D-molecular geometry. These numerical variables are derived from the elements *h*_*ij*_ of the Molecular Influence matrix (**H**), obtained through the values of atomic Cartesian coordinates. The diagonal elements of **H** (*h*_*ii*_) are called leverages, and are considered to represent the influence of each atom on the whole shape of the molecule. For instance, the mantle atoms always have higher *h*_*ii*_ values than atoms near the molecule center, while each off-diagonal element *h*_*ij*_ represents the degree of accessibility of the *j*th atom to interactions with the *i*th one. Charge descriptors are electronic descriptors defined in terms of atomic charges and used to describe electronic aspects both of the whole molecule and of particular regions, such as atoms, bonds, and molecular fragments. Charge descriptors can be considered among quantum chemical descriptors [61]. Electrical charges in the molecule are the driving force of electrostatic interactions, and it is well known that local electron densities or charges play a fundamental role in many chemical reactions, physicochemical properties and receptor-ligand binding affinity.

BCUT descriptors are the eigenvalues of a modified connectivity matrix, the Burden matrix (**B**) [62]. The ordered sequence of the **n** smallest eigenvalues of **B** has been proposed as a molecular descriptor based on the assumption that the lowest eigenvalues contain contributions from all the atoms and thus reflect the molecular topology. The BCUT descriptors are an extension of the Burden eigenvalues and consider three classes of matrices, whose diagonal elements account for atomic charge related values, atomic polarizability related values and atomic H bond abilities. Geometrical descriptors are a different kind of conformationally dependent parameters based on the molecular geometry. Reliable values are obtained if reliable conformations are previously calculated [42]. A Radial Distribution Function (RDF) [60] of an ensemble of atoms can be interpreted as the probability distribution of finding an atom in a spherical volume of certain radius, also incorporating different atomic properties, in order to differentiate the contribution of each atom to the property under study.

The QSAR models presented in Eqs. 1–10 for data sets A-G result specific to each group of Tacrine derivatives used and to each AChE source. An attempt to model the complete set of molecules (combining data sets A-G) with a single model would result in a deteriorated statistics. Furthermore, this attempt of increasing the number of experimental observations is not completely justified, as different biochemical conditions are employed for measuring the inhibitory potency. We believe that the QSAR obtained here should acceptably work in an applicability domain defined by the range of descriptor variations for each model, and would be valid for predicting structures whose experimental activities lay close to the experimental range of the training set.

## Conclusions

In this work, we present ten useful structure-activity models to analyze the activity of Acetylcholinesterase inhibitors. The proposed QSAR models highlight parallelisms between the molecular structure and the AD inhibitory activity. The importance of this study relies on the fact that new Tacrine related inhibitors are used, which have been measured under different experimental conditions and using different AChE sources. Despite of this limitation, such models show appropriate predictive capability, and the scarce amount of observations available on each data set is successfully analyzed by means of the linear methodology of the Replacement Method approach. Nevertheless, whenever newer experimental information on Tacrine-like compounds are available, further QSAR studies are encouraged on larger data sets.

## Additional files

Additional file 1:
**Molecular structures of Tacrine derivatives analyzed.** Structure 1 is Tacrine. Additional file 2: Table S1. Experimental and predicted log_10_ *IC*_50_ for bAChE inhibitory activity in data set A. Numerical values of the involved descriptors are also given. **Table S2.** Experimental and predicted log_10_ *IC*_50_ for bAChE and hAChE inhibitory activities in data set B. Numerical values of the involved descriptors are also given. **Table S3.** Experimental and predicted log_10_ *IC*_50_ for EeAChE and hAChE inhibitory activities in data set C. Numerical values of the involved descriptors are also given. **Table S4.** Experimental and predicted log_10_ *IC*_50_ for bAChE inhibitory activity in data set D. Numerical values of the involved descriptors are also given. **Table S5.** Experimental and predicted log_10_ *IC*_50_ for hAChE inhibitory activity in data set E. Numerical values of the involved descriptors are also given. **Table S6.** Experimental and predicted log_10_ *IC*_50_ for bAChE and EeAChE inhibitory activities in data set F. Numerical values of the involved descriptors are also given. **Table S7.** Experimental and predicted log_10_ *IC*_50_ for hAChE inhibitory activity in data set G. Numerical values of the involved descriptors are also given. Additional file 3:
**Predicted log**_**10**_ ***IC***_**50**_ **as the function of experimental values for data set A-G.** A: Data set A (bAChE**)**; B: Data set B (bAChE); C: Data set B (hAChE); D: Data set C (EeAChE); E: Data set C (hAChE); F: Data set D (bAChE); G: Data set E (hAChE); H: Data set F (bAChE); I: Data set F (EeAChE); J: Data set G (hAChE). Additional file 4: Figure S1A. Dispersion plot of residuals for data set A (bAChE). **Figure S1B.** Dispersion plot of residuals for data set B (bAChE). **Figure S1C.** Dispersion plot of residuals for data set B (hAChE). **Figure S1D.** Dispersion plot of residuals for data set C (EeAChE). **Figure S1E.** Dispersion plot of residuals for data set C (hAChE). **Figure S1F.** Dispersion plot of residuals for data set D (bAChE). **Figure S1G.** Dispersion plot of residuals for data set E (hAChE). **Figure S1H.** Dispersion plot of residuals for data set F (bAChE). **Figure S1I.** Dispersion plot of residuals for data set F (EeAChE). **Figure S1J.** Dispersion plot of residuals for data set G (hAChE).
