# Supplementary material for: QSAR analysis on tacrine-related acetylcholinesterase inhibitors
Source: J Biomed Sci. 2014 Sep 20;21(1):84. doi: 10.1186/s12929-014-0084-0 (PMC4177578; doi:10.1186/s12929-014-0084-0)
Supplement: Additional file 1: — Molecular structures of Tacrine derivatives analyzed. Structure 1 is Tacrine. [file 12929_2014_84_MOESM1_ESM.doc]

Figure 1. Molecular structures of Tacrine derivatives analyzed. Structure 1 is Tacrine.

**1**

|  |  | R | n |
| --- | --- | --- | --- |
|  | **2** | H | 1 |
|  | **3** | *p*-CH_3_ | 1 |
|  | **4** | *p*-Cl | 1 |
|  | **5** | *p*-CN | 1 |
|  | **6** | *p*-OCH_3_ | 1 |
|  | **7** | *m*-NO_2_ | 1 |
|  | **8** | H | 0 |

|  |  | R | n |
| --- | --- | --- | --- |
|  | **9** | H | 0 |
|  | **10** | H | 1 |
|  | **11** | H | 2 |
|  | **12** | *p*-CH_3_ | 0 |
|  | **13** | *p*-CH_3_ | 1 |
|  | **14** | *p*-CH_3_ | 2 |

|  |  | R | n | n' |
| --- | --- | --- | --- | --- |
|  | **15** | Cl | 0 | 6 |
|  | **16** | Cl | 0 | 7 |
|  | **17** | Cl | 0 | 8 |
|  | **18** | Cl | 0 | 9 |
|  | **19** | Cl | 0 | 10 |
|  | **20** | H | 2 | 4 |
|  | **21** | H | 2 | 5 |
|  | **22** | H | 2 | 6 |
|  | **23** | H | 2 | 7 |
|  | **24** | H | 2 | 8 |

|  |  | R_1_ | R_2_ | n |
| --- | --- | --- | --- | --- |
|  | **25**  **26** | Et  CH_3_ | Cl  H | 0  2 |
|  |  |  |  |  |

**27**  **28**

|  |  | **29** | **30** | **31** | **32** | **33** |
| --- | --- | --- | --- | --- | --- | --- |
|  | R | C-H | C-F | C-Me | C-OMe | N |

|  |  | **34** | **35** | **36** | **37** |
| --- | --- | --- | --- | --- | --- |
|  | R | H | F | Me | OMe |

|  |  | **38** | **40** |
| --- | --- | --- | --- |
|  | R | C-OMe | N |

|  |  | **39** | **41** |
| --- | --- | --- | --- |
|  | R | C-H | N |

|  |  | **42** | **43** | **44** | **45** |
| --- | --- | --- | --- | --- | --- |
|  | R | H | F | Me | OMe |

|  |  | R_1_ | R_2_ | R_3_ |
| --- | --- | --- | --- | --- |
|  | **46** | OMe | (CH_2_)_2_ | H |
|  | **47** | OMe | (CH_2_)_3_ | H |
|  | **48** | H | (CH_2_)_2_ | CO |

|  |  | R |
| --- | --- | --- |
|  | **49** | (CH_2_)_2_ |
|  | **50** | (CH_2_)_3_ |
|  | **51** | CH_3_N(CH_2_)_2_ |

|  |  | R |
| --- | --- | --- |
|  | **52** | (CH_2_)_3_ |
|  | **53** | (CH_2_)_4_ |

**54**

|  |  | R_1_ | R_2_ | n |
| --- | --- | --- | --- | --- |
|  | **55** | H | **A** | 6 |
|  | **56** | H | **A** | 7 |
|  | **57** | H | **A** | 8 |
|  | **58** | H | **A** | 9 |
|  | **59** | H | **A** | 10 |
|  | **60** | H | **B** | 7 |
|  | **61** | H | **C** | 7 |
|  | **62** | Cl | **C** | 7 |
|  | **63** | Cl | **D** | 7 |
|  | **64** | Cl | **E** | 7 |
|  | **65** | Cl | **F** | 7 |

**66**

**67**

|  |  | R_1_ | R_2_ |
| --- | --- | --- | --- |
|  | **68** | H | N(CH_3_)_2_ |
|  | **69** | H | **A** |
|  | **70** | H | **B** |
|  | **71** | H | **C** |
|  | **72** | Ph | N(CH_3_)_2_ |
|  | **73** | H | OCH_3_ |
|  | **74** | H | OEt |
|  | **75** | Ph | OCH_3_ |
|  | **76** | H | Cl |
|  | **77** | Ph | Cl |

|  |  | R |
| --- | --- | --- |
|  | **78** | OCH_3_ |
|  | **79** | Cl |

|  |  | R | n | n' |
| --- | --- | --- | --- | --- |
|  | **80** | Cl | 0 | 6 |
|  | **81** | Cl | 0 | 7 |
|  | **82** | Cl | 0 | 8 |
|  | **83** | Cl | 0 | 9 |
|  | **84** | Cl | 0 | 10 |
|  | **85** | H | 2 | 4 |
|  | **86** | H | 2 | 5 |
|  | **87** | H | 2 | 6 |
|  | **88** | H | 2 | 7 |
|  | **89** | H | 2 | 8 |

|  |  | R_1_ | R_2_ | n |
| --- | --- | --- | --- | --- |
|  | **90** | O | H | 2 |
|  | **91** | O | H | 3 |
|  | **92** | O | Cl | 2 |
|  | **93** | O | Cl | 3 |
|  | **94** | H,H | H | 2 |
|  | **95** | H,H | H | 3 |
|  | **96** | H,H | Cl | 2 |
|  | **97** | H,H | Cl | 3 |
