# Supplementary material for: QSAR analysis on tacrine-related acetylcholinesterase inhibitors
Source: J Biomed Sci. 2014 Sep 20;21(1):84. doi: 10.1186/s12929-014-0084-0 (PMC4177578; doi:10.1186/s12929-014-0084-0)
Supplement: Additional file 2: Table S1. — Experimental and predicted log10 IC 50 for bAChE inhibitory activity in data set A. Numerical values of the involved descriptors are also given. Table S2. Experimental and predicted log10 IC 50 for bAChE and hAChE inhibitory activities in data set B. Numerical values of the involved descriptors are also given. Table S3. Experimental and predicted log10 IC 50 for EeAChE and hAChE inhibitory activities in data set C. Numerical values of the involved descriptors are also given. Table S4. Experimental and predicted log10 IC 50 for bAChE inhibitory activity in data set D. Numerical values of the involved descriptors are also given. Table S5. Experimental and predicted log10 IC 50 for hAChE inhibitory activity in data set E. Numerical values of the involved descriptors are also given. Table S6. Experimental and predicted log10 IC 50 for bAChE and EeAChE inhibitory activities in data set F. Numerical values of the involved descriptors are also given. Table S7. Experimental and predicted log10 IC 50 for hAChE inhibitory activity in data set G. Numerical values of the involved descriptors are also given. [file 12929_2014_84_MOESM2_ESM.doc]

**Table S1.** Experimental and predicted for bAChE inhibitory activity in data set A. Numerical values of the involved descriptors are also given.

| **Compound ID** | **Exp. bAChE** | **Eq. 2** | ***Mor*06*v*** | ***G*1*u*** |
| --- | --- | --- | --- | --- |
| **1** | -6.886 | -7.002 | 0.649 | 0.171 |
| **2**^t^ | -5.807 | -5.560 | 0.172 | 0.150 |
| **3** | -5.740 | -5.541 | 0.240 | 0.148 |
| **4** | -5.714 | -5.934 | 0.528 | 0.150 |
| **5** | -5.460 | -5.498 | 0.156 | 0.149 |
| **6** | -6.061 | -5.967 | 0.304 | 0.156 |
| **7** | -5.724 | -5.800 | 0.060 | 0.158 |
| **8** | -5.409 | -5.326 | -0.136 | 0.152 |
| **9** | -4.924 | -4.952 | -0.534 | 0.153 |
| **10** | -6.085 | -5.505 | 0.077 | 0.151 |
| **11** | -4.678 | -5.043 | -0.277 | 0.149 |
| **12**^t^ | -4.863 | -4.929 | -0.428 | 0.150 |
| **13** | -5.047 | -5.388 | 0.094 | 0.148 |
| **14** | -5.870 | -5.642 | 0.037 | 0.155 |

^t^ Test set molecule

**Table S2.** Experimental and predicted for bAChE and hAChE inhibitory activities in data set B. Numerical values of the involved descriptors are also given.

| **Compound ID** | **Exp. bAChE** | **Eq. 3** | **Exp. hAChE** | **Eq. 4** | ***GATS*2*e*** | ***R*5*e*** | ***Qmean*** | ***R*3*m^+^*** |
| --- | --- | --- | --- | --- | --- | --- | --- | --- |
| **15** | -7.690 | -7.838 | -7.717 | -7.652 | 0.791 | 1.324 | 0.086 | 0.049 |
| **16**^t,u^ | -7.987 | -7.298 | -7.738 | -8.074 | 0.788 | 1.199 | 0.085 | 0.040 |
| **17** | -7.983 | -7.688 | -8.153 | -7.891 | 0.786 | 1.283 | 0.084 | 0.048 |
| **18** | -7.620 | -7.648 | -7.604 | -7.500 | 0.784 | 1.271 | 0.084 | 0.059 |
| **19** | -7.028 | -7.390 | -7.301 | -7.571 | 0.783 | 1.212 | 0.084 | 0.057 |
| **20** | -7.318 | -7.179 | -7.780 | -7.794 | 0.833 | 1.240 | 0.086 | 0.045 |
| **21** | -7.622 | -7.331 | -8.016 | -8.114 | 0.831 | 1.271 | 0.086 | 0.036 |
| **22**^t^ | -7.511 | -7.112 | -7.955 | -7.611 | 0.829 | 1.219 | 0.085 | 0.053 |
| **23**^u^ | -7.524 | -7.367 | -7.842 | -7.682 | 0.827 | 1.273 | 0.085 | 0.051 |
| **24** | -7.467 | -7.476 | -7.854 | -8.135 | 0.826 | 1.296 | 0.083 | 0.044 |
| **25** | -5.000 | -4.858 | -5.000 | -5.080 | 0.885 | 0.800 | 0.106 | 0.064 |
| **26** | -5.000 | -5.199 | - | - | 0.948 | 0.971 | - | - |
| **27** | -5.201 | -5.281 | -4.491 | -4.474 | 1.390 | 1.655 | 0.128 | 0.018 |
| **28** | -8.242 | -8.441 | -8.080 | -8.128 | 0.584 | 1.147 | 0.074 | 0.070 |

^t^ Test set molecule for bAChE; ^u^ Test set molecule for hAChE

**Table S3.** Experimental and predicted for EeAChE and hAChE inhibitory activities in data set C. Numerical values of the involved descriptors are also given.

| **Compound ID** | **Exp.**  **EeAChE** | **Eq. 5** | **Exp.**  **hAChE** | **Eq. 6** | ***R*5*e^+^*** | ***nNHR*** | ***X*1*A*** |
| --- | --- | --- | --- | --- | --- | --- | --- |
| **1** | -6.745 | -7.023 | -6.824 | -6.689 | 0.053 | 0 | 0.433 |
| **27** | -4.959 | -5.025 | - | - | 0.024 | 1 | - |
| **29** | -6.495 | -6.200 | -6.149 | -6.007 | 0.024 | 1 | 0.420 |
| **30** | -6.268 | -6.200 | -5.796 | -5.955 | 0.024 | 1 | 0.419 |
| **31**^t^ | -6.638 | -6.200 | -6.174 | -5.955 | 0.029 | 1 | 0.419 |
| **32** | -6.658 | -6.616 | -6.432 | -6.165 | 0.026 | 1 | 0.423 |
| **33** | -6.222 | -6.367 | -5.553 | -6.007 | 0.037 | 1 | 0.420 |
| **34** | -7.097 | -7.282 | -6.921 | -6.899 | 0.037 | 1 | 0.437 |
| **35** | -7.284 | -7.282 | -6.721 | -6.847 | 0.035 | 1 | 0.436 |
| **36**^u^ | -7.041 | -7.116 | -6.770 | -6.847 | 0.036 | 1 | 0.436 |
| **37**^t^ | -7.347 | -7.199 | -6.959 | -7.004 | 0.036 | 0 | 0.439 |
| **38** | -6.060 | -5.608 | - | - | 0.039 | 0 | - |
| **39** | -5.523 | -5.275 | - | - | 0.032 | 0 | - |
| **40** | -6.086 | -5.857 | - | - | 0.039 | 0 | - |
| **41** | -5.854 | -5.857 | - | - | 0.024 | 0 | - |
| **42**^t^ | -4.310 | -4.609 | - | - | 0.023 | 0 | - |
| **43** | -4.155 | -4.525 | - | - | 0.023 | 0 | - |
| **44** | -4.284 | -4.525 | - | - | 0.033 | 0 | - |
| **45** | -5.387 | -5.358 | - | - | 0.029 | 0 | - |

^t^ Test set molecule for EeAChE; ^u^ Test set molecule for hAChE

**Table S4.** Experimental and predicted for bAChE inhibitory activity in data set D. Numerical values of the involved descriptors are also given.

| **Compound ID** | **Exp. bAChE** | **Eq. 7** | ***Mor*09*u*** | ***R2m^+^*** |
| --- | --- | --- | --- | --- |
| **1** | -6.777 | -6.717 | 0.166 | 0.031 |
| **46** | -7.000 | -6.851 | 0.400 | 0.026 |
| **47**^t^ | -7.602 | -8.017 | 1.195 | 0.031 |
| **48** | -6.298 | -6.196 | -0.195 | 0.029 |
| **49** | -8.000 | -7.870 | -0.423 | 0.090 |
| **50** | -7.699 | -7.639 | 0.005 | 0.066 |
| **51** | -8.553 | -8.707 | 0.214 | 0.091 |
| **52** | -5.522 | -5.827 | -0.462 | 0.028 |
| **53** | -7.032 | -6.934 | 0.440 | 0.027 |
| **54** | -7.721 | -7.861 | 1.046 | 0.032 |

^t^ Test set molecule

**Table S5.** Experimental and predicted for hAChE inhibitory activity in data set E. Numerical values of the involved descriptors are also given.

| **Compound ID** | **Exp. hAChE** | **Eq. 8** | ***BELm*2** | ***Mor*27*e*** |
| --- | --- | --- | --- | --- |
| **1** | -6.602 | -6.460 | 1.917 | -0.259 |
| **55** | -7.162 | -6.701 | 1.927 | 0.022 |
| **56** | -7.352 | -7.525 | 1.936 | 0.223 |
| **57** | -7.783 | -8.127 | 1.942 | 0.191 |
| **58**^t^ | -7.469 | -8.081 | 1.948 | -0.016 |
| **59** | -7.547 | -7.563 | 1.988 | -0.450 |
| **60** | -6.548 | -6.659 | 2.068 | -0.266 |
| **61** | -7.857 | -7.689 | 2.049 | 0.133 |
| **62** | -8.979 | -8.638 | 2.030 | 0.042 |
| **63** | -7.917 | -8.266 | 1.964 | -0.427 |
| **64** | -6.509 | -6.561 | 1.964 | -0.541 |
| **65**^t^ | -6.588 | -6.253 | 2.067 | -0.678 |
| **66** | -6.342 | -6.571 | 1.857 | -0.200 |
| **67** | -9.092 | -8.929 | 2.108 | 0.095 |

^t^ Test set molecule

**Table S6.** Experimental and predicted for bAChE and EeAChE inhibitory activities in data set F. Numerical values of the involved descriptors are also given.

| **Compound ID** | **Exp.**  **bAChE** | **Eq. 9** | **Exp.**  **EeAChE** | **Eq. 10** | ***AMW*** | ***MEcc*** | ***E*2*u*** | ***HATS*6*m*** |
| --- | --- | --- | --- | --- | --- | --- | --- | --- |
| **1** | -6.963 | -7.169 | -7.569 | -7.485 | 7.230 | 1.000 | 0.487 | 0.068 |
| **68** | -7.000 | -6.629 | -7.854 | -7.608 | 7.160 | 1.000 | 0.476 | 0.087 |
| **69** | -6.796 | -6.726 | -7.367 | -7.407 | 6.990 | 0.999 | 0.451 | 0.090 |
| **70** | -6.000 | -6.081 | -7.602 | -7.687 | 7.980 | 1.000 | 0.459 | 0.092 |
| **71** | -5.815 | -5.591 | -7.523 | -7.543 | 7.310 | 0.999 | 0.482 | 0.093 |
| **72**^u^ | -5.357 | -5.638 | -7.046 | -7.217 | 7.710 | 1.000 | 0.519 | 0.075 |
| **73**^u^ | -5.796 | -5.965 | -7.222 | -7.052 | 7.450 | 1.000 | 0.496 | 0.070 |
| **74**^t^ | -6.000 | -6.325 | -7.301 | -7.451 | 7.680 | 0.999 | 0.454 | 0.121 |
| **75**^t^ | -5.301 | -5.125 | -7.097 | -7.080 | 8.920 | 1.000 | 0.512 | 0.176 |
| **76** | -4.000 | -4.289 | -5.301 | -5.296 | 8.590 | 0.999 | 0.387 | 0.306 |
| **77** | -4.000 | -3.865 | -4.523 | -4.590 | 7.680 | 0.999 | 0.533 | 0.081 |
| **78** | -5.301 | -5.125 | -6.456 | -6.754 | 8.530 | 0.999 | 0.495 | 0.142 |
| **79** | -4.000 | -3.949 | -6.456 | -6.146 | 6.840 | 1.000 | 0.514 | 0.055 |

^t^ Test set molecule for bAChE; ^u^ Test set molecule for EeAChE

**Table S7.** Experimental and predicted for hAChE inhibitory activity in data set G. Numerical values of the involved descriptors are also given.

| **Compound ID** | **Exp. hAChE** | **Eq. 11** | ***MPC09*** | ***MATS1m*** | ***RDF020m*** |
| --- | --- | --- | --- | --- | --- |
| **1** | -6.688 | -6.617 | 355 | 0.939 | 0.989 |
| **27** | -5.000 | -5.176 | 356 | 0.937 | 0.970 |
| **28** | -8.080 | -8.210 | 357 | 0.935 | 0.993 |
| **54** | -7.936 | -7.719 | 358 | 0.933 | 1.011 |
| **80** | -7.717 | -8.004 | 359 | 0.931 | 1.028 |
| **81**^t^ | -7.738 | -7.717 | 334 | 0.931 | 1.021 |
| **82** | -8.153 | -7.685 | 334 | 0.929 | 1.035 |
| **83** | -7.604 | -7.624 | 335 | 0.927 | 1.056 |
| **84** | -7.301 | -7.556 | 336 | 0.926 | 1.093 |
| **85** | -7.780 | -7.865 | 337 | 0.924 | 1.108 |
| **86** | -8.016 | -7.793 | 241 | 0.915 | 1.125 |
| **87** | -7.955 | -7.749 | 235 | 0.913 | 1.229 |
| **88** | -7.842 | -7.882 | 255 | 0.925 | 1.106 |
| **89**^t^ | -7.854 | -7.802 | 249 | 0.923 | 1.209 |
| **90** | -8.394 | -8.549 | 229 | 0.907 | 1.139 |
| **91** | -9.056 | -9.109 | 223 | 0.906 | 1.151 |
| **92** | -9.174 | -9.023 | 243 | 0.918 | 1.116 |
| **93** | -9.569 | -9.577 | 237 | 0.916 | 1.193 |
| **94** | -8.290 | -8.174 | 66 | 0.919 | 0.351 |
| **95**^t^ | -8.666 | -8.253 | 77 | 0.943 | 0.328 |
| **96** | -8.585 | -8.702 | 269 | 0.907 | 0.739 |
| **97** | -8.975 | -9.098 | 137 | 0.915 | 0.748 |

^t^ Test set molecule
