# Supplementary material for: QSAR analysis on tacrine-related acetylcholinesterase inhibitors
Source: J Biomed Sci. 2014 Sep 20;21(1):84. doi: 10.1186/s12929-014-0084-0 (PMC4177578; doi:10.1186/s12929-014-0084-0)
Supplement: Additional file 3: — Predicted log 10 IC 50 as the function of experimental values for data set A-G. A: Data set A (bAChE); B: Data set B (bAChE); C: Data set B (hAChE); D: Data set C (EeAChE); E: Data set C (hAChE); F: Data set D (bAChE); G: Data set E (hAChE); H: Data set F (bAChE); I: Data set F (EeAChE); J: Data set G (hAChE). [file 12929_2014_84_MOESM3_ESM.doc]

Figure 2A. Predicted as function of experimental values for data set A (bAChE).

Figure 2B. Predicted as function of experimental values for data set B (bAChE).

Figure 2C. Predicted as function of experimental values for data set B (hAChE).

Figure 2D. Predicted as function of experimental values for data set C (EeAChE).

Figure 2E. Predicted as function of experimental values for data set C (hAChE).

Figure 2F. Predicted as function of experimental values for data set D (bAChE).

Figure 2G. Predicted as function of experimental values for data set E (hAChE).

Figure 2H. Predicted as function of experimental values for data set F (bAChE).

Figure 2I. Predicted as function of experimental values for data set F (EeAChE).

Figure 2J. Predicted as function of experimental values for data set G (hAChE).
