# Supplementary material for: QSAR analysis on tacrine-related acetylcholinesterase inhibitors
Source: J Biomed Sci. 2014 Sep 20;21(1):84. doi: 10.1186/s12929-014-0084-0 (PMC4177578; doi:10.1186/s12929-014-0084-0)
Supplement: Additional file 4: Figure S1A. — Dispersion plot of residuals for data set A (bAChE). Figure S1B. Dispersion plot of residuals for data set B (bAChE). Figure S1C. Dispersion plot of residuals for data set B (hAChE). Figure S1D. Dispersion plot of residuals for data set C (EeAChE). Figure S1E. Dispersion plot of residuals for data set C (hAChE). Figure S1F. Dispersion plot of residuals for data set D (bAChE). Figure S1G. Dispersion plot of residuals for data set E (hAChE). Figure S1H. Dispersion plot of residuals for data set F (bAChE). Figure S1I. Dispersion plot of residuals for data set F (EeAChE). Figure S1J. Dispersion plot of residuals for data set G (hAChE). [file 12929_2014_84_MOESM4_ESM.doc]

**Figure S1A.** Dispersion plot of residuals for data set A (bAChE).

**Figure S1B.** Dispersion plot of residuals for data set B (bAChE).

**Figure S1C.** Dispersion plot of residuals for data set B (hAChE).

**Figure S1D.** Dispersion plot of residuals for data set C (EeAChE).

**Figure S1E.** Dispersion plot of residuals for data set C (hAChE).

**Figure S1F.** Dispersion plot of residuals for data set D (bAChE).

**Figure S1G.** Dispersion plot of residuals for data set E (hAChE).

**Figure S1H.** Dispersion plot of residuals for data set F (bAChE).

**Figure S1I.** Dispersion plot of residuals for data set F (EeAChE).

**Figure S1J.** Dispersion plot of residuals for data set G (hAChE).
